# Effects of Dietary Wolfberry Sprout Stems and Leaves on the Rumen Bacterial Community of Fattening Tan Sheep and Its Association with Fermentation and Growth Traits

**DOI:** 10.3390/ani16213430

**Published:** 2026-10-30

**Authors:** Jin Wang, Huiming Zhang, Yu Fan, Qingmin Li, Jingsong Sun, Jifeng Ma, Qing Ma, Yuxiang Zhou

**Affiliations:** 1 College of Animal Science and Technology, Ningxia University, Yinchuan 750021, China; wangjin8208@163.com (J.W.); z1137366741@163.com (H.Z.); fanyu0128@126.com (Y.F.); liqingmin615@163.com (Q.L.); 2 Institute of Animal Science, Ningxia Academy of Agriculture and Forestry Sciences, Yinchuan 750002, China; maqing1973@126.com; 3 Liaoyuan Animal Husbandry Technology Extension Service Center, Liaoyuan 136200, China; s17611010109@126.com

**Keywords:** 16S rRNA gene, rumen bacteria, Spearman correlation, Tan sheep, wolfberry sprout stems and leaves

## Abstract

Wolfberry (*Lycium barbarum*) sprouts are harvested on a large scale in Ningxia, and the residual tender stems and leaves constitute an abundant but largely unused by-product. This experiment was conducted to investigate the effects of dietary supplementation with different proportions of wolfberry (*Lycium barbarum*) sprout stems and leaves (WSSLs) on rumen fermentation and the rumen bacterial community of fattening Tan sheep, and to reveal the relationships between rumen bacteria and fermentation and growth traits. Forty-eight healthy 3-month-old weaned Tan rams with similar body weight ((22.75 ± 0.52) kg) were randomly divided into four groups of 12 sheep; the control group (G0) was fed a basal diet, while the G4, G8 and G12 groups were fed basal diets in which 4%, 8% and 12% of the corn straw was replaced by WSSLs. The adaptation period lasted 10 days and the experimental period 60 days. At the end of the experiment, six sheep per group (24 in total) were slaughtered and rumen contents were collected for fermentation measurements, and the V3–V4 region of the bacterial 16S rRNA gene was sequenced on the Illumina NovaSeq platform. Ruminal pH, ammonia nitrogen (NH3-N) and microbial crude protein (MCP) did not differ among groups (*p* > 0.05), whereas total volatile fatty acid (VFA) concentration differed (30.15–56.27 mmol/L; *p* = 0.025), together with acetate (*p* = 0.032) and butyrate (*p* = 0.010); these VFA differences were driven mainly by the high and variable values in the control group and showed no dose–response. Average daily gain was higher in G8 than in G0 and G4 (*p* = 0.010), and live weight before slaughter and carcass weight increased with the dietary WSSL level (*p* = 0.011 and 0.013). Seventeen phyla and 210 genera were identified, with Bacteroidota and Bacillota dominant; the relative abundance of Bacteroidota decreased while that of Bacillota tended to increase with the WSSL level, and *Xylanibacter* was the most abundant genus (15.85–18.18%). Alpha diversity indices did not differ among groups (*p* > 0.05), and principal coordinate analysis (PCoA) based on Bray–Curtis distance showed limited separation (Adonis R^2^ = 0.157, *p* = 0.002). Nine genera showed nominal between-group variation (uncorrected *p* < 0.05); however, after Benjamini–Hochberg FDR correction across all 210 genera tested, none remained significant (all q = 1.00). *Howardella* was negatively correlated with acetate, propionate, butyrate and total VFA (*p* < 0.05), U29-B03 was positively correlated with live weight before slaughter and carcass weight (*p* < 0.05), and bacteria of Christensenellaceae were positively correlated with propionate, butyrate and total VFA (*p* < 0.05); none of these associations survived FDR correction. Dietary supplementation with 4–12% WSSLs did not depress growth performance and did not significantly alter the abundance of any individual taxon after correction for multiple testing; it moderately alters rumen bacterial community composition, and WSSLs can be used safely at inclusion levels of 4–12% as an unconventional feed resource for fattening Tan sheep.

## Simple Summary

Wolfberry (*Lycium barbarum*) is widely grown in Ningxia, China, and harvesting its young edible sprouts generates large amounts of tender stems and leaves that are usually discarded. We tested whether these sprout stems and leaves could be fed to fattening Tan sheep without harming rumen function, and how they affected rumen bacteria. Replacing 4%, 8%, or 12% of the corn straw in the diet with this by-product did not change ruminal acidity or microbial protein, nor the overall diversity of rumen bacteria; total volatile fatty acid concentration differed among groups, but the difference was driven by the variable control animals and lacked a dose–response. It did, however, shift bacterial community composition: the proportion of Bacteroidota fell while that of Bacillota rose, and nine bacterial genera changed in abundance. Several of these genera were closely related to fatty acid formation and to live and carcass weights. We conclude that wolfberry sprout stems and leaves are a safe, unconventional feed for fattening sheep; the rumen bacterial changes observed were nominally associated with fermentation and growth traits but need further confirmation. Including this by-product in the diet is also a practical way to recycle an agricultural residue.

## 1. Introduction

The rumen is a unique anaerobic fermentation chamber of ruminants, in which a dense and complex microbial community degrades fibrous material and non-protein nitrogen that the host cannot digest and converts them into volatile fatty acids (VFAs) and microbial protein, supplying approximately 70% of the host’s energy and most of its available nitrogen [1]. A global survey of rumen microbiota across a wide geographical range revealed a relatively stable core microbiome, with diet composition being the strongest determinant of community structure [2]. When ruminants are switched from forage to high-concentrate diets, the rumen microbiota undergoes predictable succession [3], and diet type together with induced ruminal acidosis can markedly alter the diversity of epimural bacteria [4]; these microbial changes are directly linked to fermentation efficiency, methane emissions and host health [1,5]. Direct manipulation of the rumen microbiota through nutritional means is therefore an important strategy for improving feed efficiency and animal performance [6,7,8].

Wolfberry (*Lycium barbarum* L.) is a characteristic crop of Ningxia, where it is planted across about 350,000 mu. Annual pruning and sprout harvesting generate large quantities of tender stems, leaves and branches (about 2 × 10^6^ t per year), most of which have long been discarded or burned [9]. Wolfberry sprout stems and leaves are rich in polysaccharides, flavonoids, polyphenols, carotenoids and betaine, and show antioxidant, anti-inflammatory and immunomodulatory activities [9,10]. In livestock and poultry production, *L. barbarum* polysaccharides improved the growth performance, slaughter performance and meat quality of lambs [10], increased nutrient apparent digestibility, energy and nitrogen metabolism and serum immune function [11], and fermented wolfberry residue enhanced the immunity and meat quality of Tan sheep by regulating immune-related genes and metabolic pathways [12,13].

The use of wolfberry by-products in ruminant diets has increased in recent years. Duan et al. [14] reported that wolfberry branches and leaves enhanced growth performance and improved the rumen microbiota of Hu sheep. Studies using black wolfberry (*Lycium ruthenicum*) branches showed that they modulated plasma biochemical indices and rumen microbiota [15] and improved muscle amino and fatty acid composition [16] in sheep, while residual black wolfberry fruit altered rumen fermentation and microbiota and increased economic returns in fattening sheep [17]. Wolfberry by-products increased rumen fermentation and digestive enzyme activity and enhanced immune and antioxidant capacity in grazing sheep [18], and replacing part of dietary maize with wolfberry residue altered fecal microbiota without affecting growth in Simmental cattle [19]. In Tan sheep, Zhang et al. [20] demonstrated that wolfberry residue promoted growth by increasing feed intake and regulating the rumen microbiome and metabolome. Within China, wolfberry residue was also found to affect the feeding behavior and nutrient digestibility of housed Tan sheep [21], and wolfberry branches and other roughages modulated production performance, meat quality and serum biochemical indices [22].

The colonization and succession of the rumen microbiota in young ruminants are highly plastic in response to diet [23], and high-throughput sequencing of the 16S rRNA gene has become a key tool for resolving rumen community structure and its links with host phenotypes [24,25]. Platforms such as QIIME 2 with DADA2 denoising [26,27], together with OTU clustering and taxonomic classification [28,29], allow fine-scale characterization of communities at the ASV/OTU level, after which differential abundance and correlation analyses [30] can connect the microbiota with fermentation and growth traits. Wolfberry sprout stems and leaves are the tender tissues remaining after edible sprouts are harvested; compared with older branches, they contain more crude protein, are less fibrous and are more palatable. However, their effects on the rumen bacterial community of fattening Tan sheep, and the links between bacterial changes and fermentation and growth traits, have not been reported. Building on our earlier finding that WSSLs improved the growth performance of Tan sheep, this study used 16S rRNA gene sequencing together with rumen fermentation measurements to characterize the response of the rumen bacterial community to increasing WSSL levels, and applied Spearman correlation analysis to identify genera associated with fermentation and growth traits, aiming to provide a theoretical basis for the value-added feed use of WSSLs and for rumen nutritional regulation in Tan sheep.

## 2. Materials and Methods

### 2.1. Experimental Site and Period

The experiment was conducted from August to December 2025 at the Nuanquan Sheep Farm of Ningxia Nongken Beef and Mutton Food Co., Ltd. (Yinchuan, China). The animal protocol was approved by the Animal Ethics Committee of Ningxia University (approval no. NXU-A-2025-324; approval date: 1 July 2025).

### 2.2. Animals and Experimental Design

A single-factor completely randomized design was used. Forty-eight healthy 3-month-old weaned Tan rams were used. The individual animal was the experimental unit for all measurements reported in this study. During the 10-day adaptation period, the proportion of WSSL was increased stepwise to the assigned level, and the full replacement level shown in Table 1 was fed throughout the 60-day experimental period. Tan rams with similar body weight ((22.75 ± 0.52) kg) and body condition were randomly assigned to four groups of 12 sheep, with three replicates of four sheep. The control group (G0) received a basal diet; in groups G4, G8 and G12, 4%, 8% and 12% of the corn straw in the basal diet was replaced with WSSL, respectively. A 10-day adaptation period was followed by a 60-day experimental period. Sheep were tethered and housed indoors, fed twice daily at 08:00 and 17:00, with free access to feed and water; routine vaccination and deworming followed farm procedures. At the end of the experiment, six sheep with body weight close to the group mean (24 in total) were selected and slaughtered, and rumen fluid was collected for fermentation measurements and 16S rRNA gene sequencing. Blinding was not applied in this study, as the four experimental diets were visibly different among groups; the trial was therefore conducted in an open-label manner. The ARRIVE 2.0 guidelines checklist is provided as Table S1.

### 2.3. Experimental Diets

WSSL obtained from the wolfberry sprout plantation base of Ningxia Lüse Shiji Agricultural Science and Technology Co., Ltd. in Hongguang Town, Helan County, Yinchuan, Ningxia, China; it consisted of the tender stems and leaves harvested after each crop of edible sprouts, naturally air-dried, ground through a 40-mesh sieve and stored. Diets were formulated to be isonitrogenous and isoenergetic according to the Nutrient Requirements of Meat Sheep (NY/T 816–2021); their composition and nutrient levels are shown in Table 1.

### 2.4. Rumen Fluid Collection and Fermentation Measurements

Before slaughter, sheep were fasted for 24 h and deprived of water for 2 h. Immediately after slaughter, the rumen was opened and about 50 g of contents was collected from different rumen regions and filtered through four layers of gauze. The liquid fraction was divided into aliquots: one was used for on-site pH measurement with a portable pH meter (Leici PHS-3C; Shanghai Leici Instrument Co., Ltd., Shanghai, China), and the rest was snap-frozen in liquid nitrogen and stored at −80 °C. Ammonia nitrogen (NH3-N) was determined by the phenol–hypochlorite colorimetric method [31], microbial crude protein (MCP) by the Lowry method with Folin-phenol reagent [32], and VFAs by gas chromatography (Agilent 7890A; Agilent Technologies, Santa Clara, CA, USA) using 2-ethylbutyric acid as the internal standard [33]. At the end of the 24h fast, live weight before slaughter was recorded. Carcass weight was recorded after removal of the skin, head, feet and internal organs, with the kidneys and perirenal fat retained. The GR value was measured as the tissue thickness between the 12th and 13th ribs, 11 cm from the dorsal midline. Average daily gain was calculated as (final body weight−initial body weight) divided by the number of days between the first and the final weighing.

### 2.5. DNA Extraction and 16S rRNA Gene Sequencing

Total microbial DNA was extracted using the CTAB/SDS method. The V3–V4 region of the 16S rRNA gene was amplified with primers 338F (5′-ACTCCTACGGGAGGCAGCAG-3′) and 806R (5′-GGACTACHVGGGTWTCTAAT-3′). Purified PCR products were sequenced on the Illumina NovaSeq platform (PE250). Raw reads were processed in QIIME 2 (version 2024), ASVs were resolved with DADA2 (version 2024), and taxonomic assignment used the Silva 138 database.

### 2.6. Predicted Functional Profiling

Metabolic functions of the rumen bacterial community were predicted with PICRUSt2 (version 2.2.0). The ASV table was normalized against predicted 16S rRNA gene copy numbers, and functions were annotated against the KEGG (KO; pathway levels 1–3), COG and MetaCyc databases. Prediction reliability was evaluated with the weighted nearest sequenced taxon index (NSTI). Relative abundances of predicted functions were compared among groups by one-way ANOVA.

### 2.7. Statistical Analysis

Differences in fermentation traits, growth and slaughter traits and bacterial relative abundances among groups were tested by one-way analysis of variance using the model Yij = μ + Ti + eij, where Yij is the observed value, μ the overall mean, Ti the fixed effect of treatment group (i = G0, G4, G8, G12) and eij the random residual error. Alpha diversity indices (ACE, Chao1, Shannon, Simpson) were calculated in Mothur (version 1.48.3). Beta diversity was assessed by PCoA based on Bray–Curtis distances, and differences among groups were tested by Adonis (vegan package, version 2.6-4; PERMANOVA, 999 permutations). Genera differing among groups were identified by one-way ANOVA followed by Duncan’s multiple range test, with p < 0.05 considered significant; because 210 genera were detected in the experimental animals, the resulting p-values were additionally adjusted by the Benjamini–Hochberg FDR procedure [30]. Relationships between the nominal differential genera and fermentation parameters and performance traits were evaluated by Spearman rank correlation on all 24 sheep, and the resulting p-values (90 genus–trait tests; Table S2) were adjusted by the Benjamini–Hochberg FDR procedure [30]. All analyses were performed in R 4.2.1 and Python 3.10.

## 3. Results

### 3.1. Rumen Fermentation Parameters

As shown in Table 2, ruminal pH ranged from 6.58 to 6.77 across groups, remaining within the physiological range (6.2–7.0) and did not differ among groups (*p* = 0.266). NH3-N (*p* = 0.608) and MCP (*p* = 0.674) were likewise unaffected by dietary WSSLs. In contrast, total VFA concentration differed among groups (30.15–56.27 mmol/L; *p* = 0.025), as did acetate (*p* = 0.032), butyrate (*p* = 0.010) and valerate (*p* = 0.039), all of which were numerically highest in G0 and lowest in G4. Propionate (*p* = 0.067) and the acetate-to-propionate ratio (*p* = 0.153) did not differ among groups. Because the between-group variances were heterogeneous (the SD in G0 was 3–5 times larger than in the WSSL groups), Welch’s heteroscedastic ANOVA was additionally applied; total VFA remained significant under this test (*p* = 0.033), as did propionate (*p* = 0.017), whereas acetate, butyrate and valerate were no longer significant (*p* = 0.078, 0.057 and 0.087, respectively). These VFA differences should therefore be interpreted with caution: they were driven mainly by the high and highly variable values in the unsupplemented control and did not follow a consistent dose–response across the 4%, 8% and 12% WSSL levels.

### 3.2. Growth Performance and Slaughter Traits

Growth performance and slaughter traits differed among treatments (Table 3). Average daily gain was higher in G8 than in G0 and G4 (*p* = 0.010), and live weight before slaughter and carcass weight increased with an increasing dietary WSSL level (*p* = 0.011 and 0.013, respectively), whereas GR value did not differ (*p* = 0.493). These results indicate that replacing 4–12% of dietary corn straw with WSSLs did not depress, and at the 8% inclusion level tended to improve, the growth performance of fattening Tan sheep.

### 3.3. Rumen Bacterial Alpha Diversity

As shown in Table 4, the ACE index was highest in G12 (694.07) and lowest in G4 (617.49), but did not differ significantly among groups (*p* > 0.05); Chao1 showed a similar pattern. Shannon indices ranged from 5.35 to 5.40 and Simpson indices were close to zero (0.01) in all groups, indicating high evenness and low dominance. Coverage was close to 1.00 in all groups, confirming sufficient sequencing depth.

### 3.4. Rumen Bacterial Community Composition

**Figure 1:**
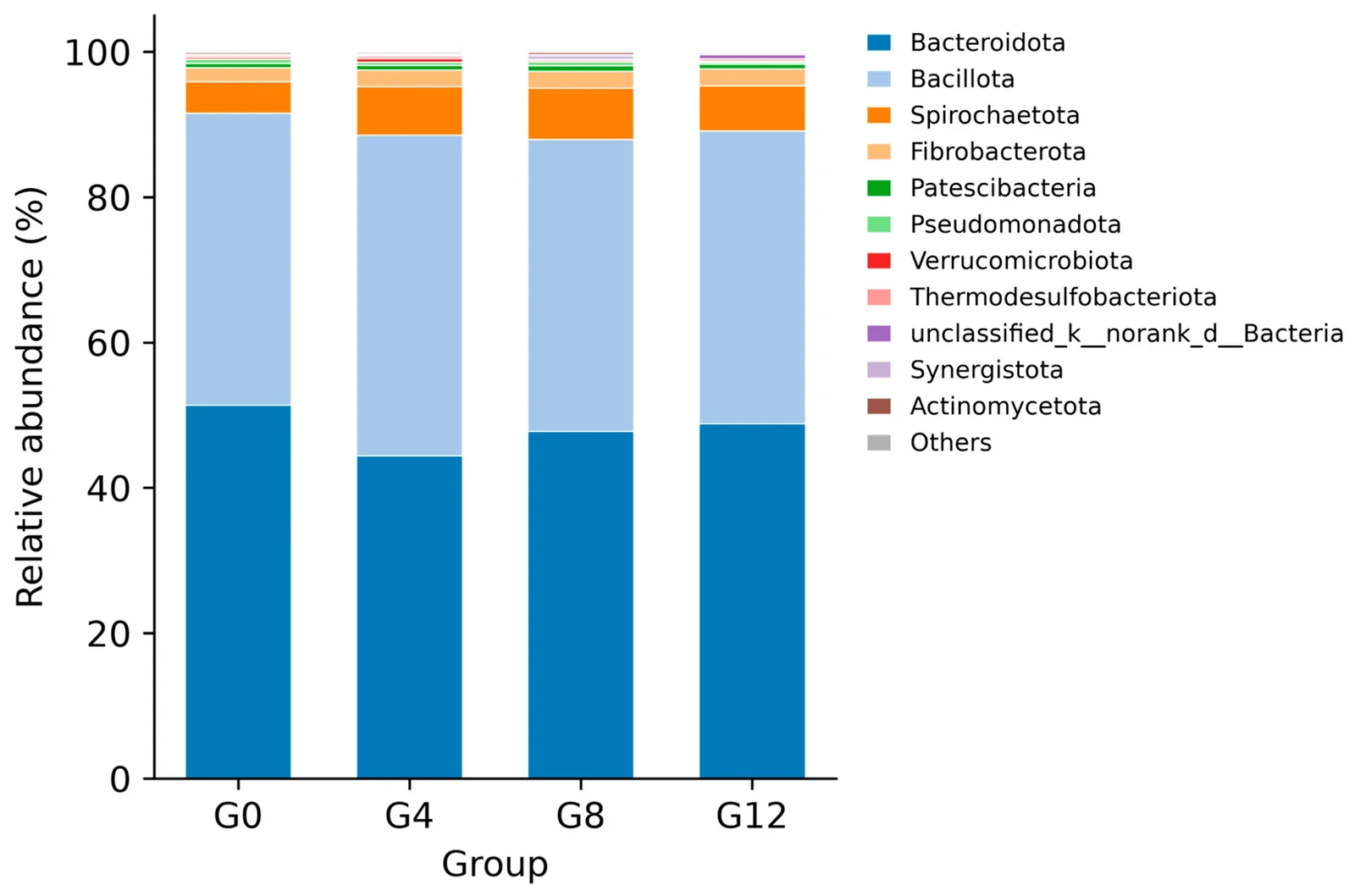
Relative abundance of rumen bacterial phyla in each group.

At the phylum level (Figure 1), 17 phyla were detected. Bacteroidota was the most abundant phylum (44.41–51.38%), highest in G0 and lowest in G4; Bacillota was the second most abundant (40.17–44.16%). Spirochaetota (4.36–7.04%) and Fibrobacterota (1.93–2.37%) were also present.

**Figure 2:**
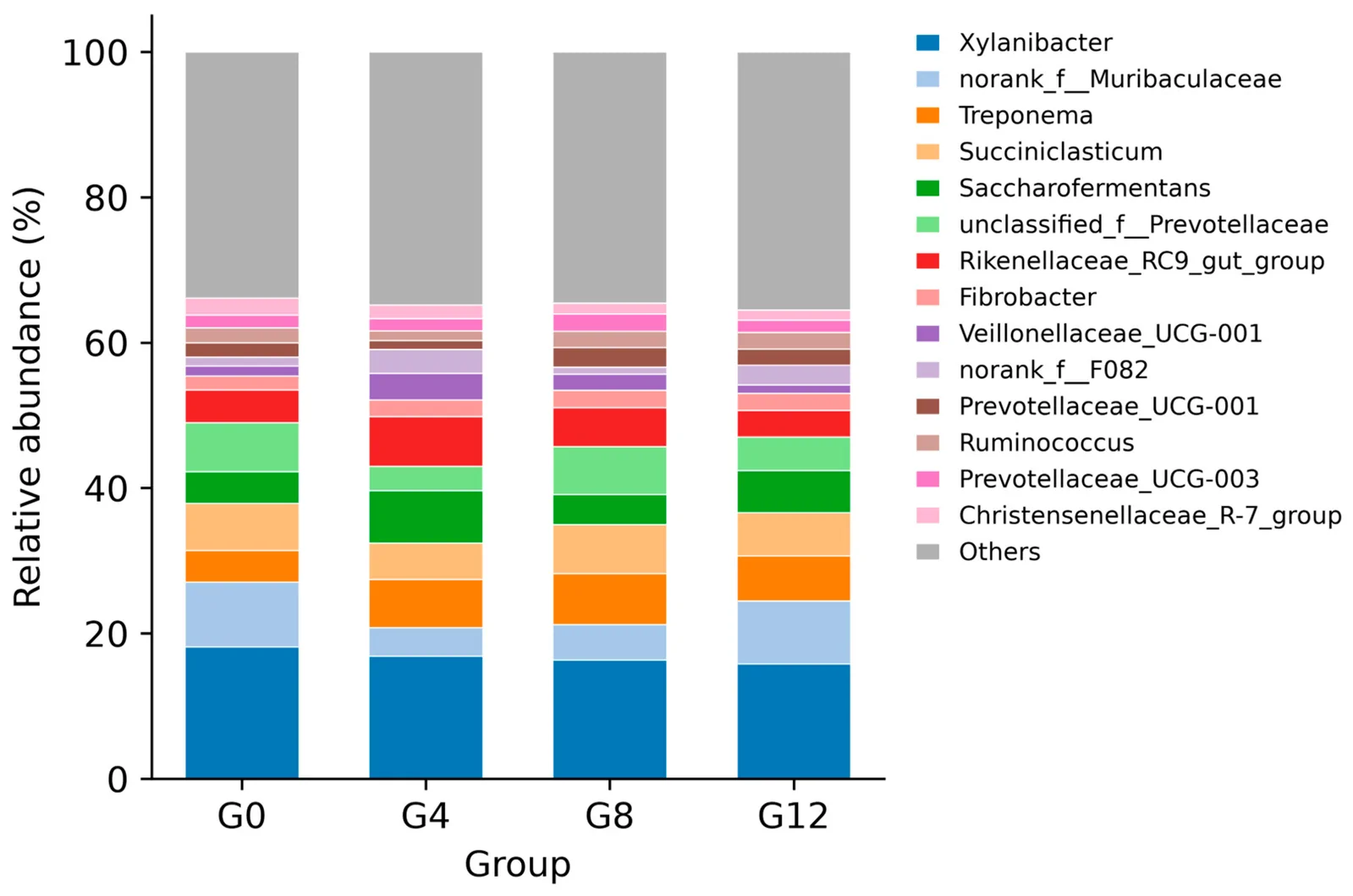
Relative abundance of rumen bacterial genera in each group.

At the genus level (Figure 2), 210 genera were identified. *Xylanibacter* was the most abundant genus (15.85–18.18%) and tended to decrease as the WSSL level increased. Other abundant genera included norank_f__Muribaculaceae (3.91–8.89%), *Treponema* (4.35–7.04%), *Succiniclasticum* (4.97–6.69%) and *Saccharofermentans* (4.18–7.24%).

### 3.5. Rumen Bacterial Beta Diversity

**Figure 3:**
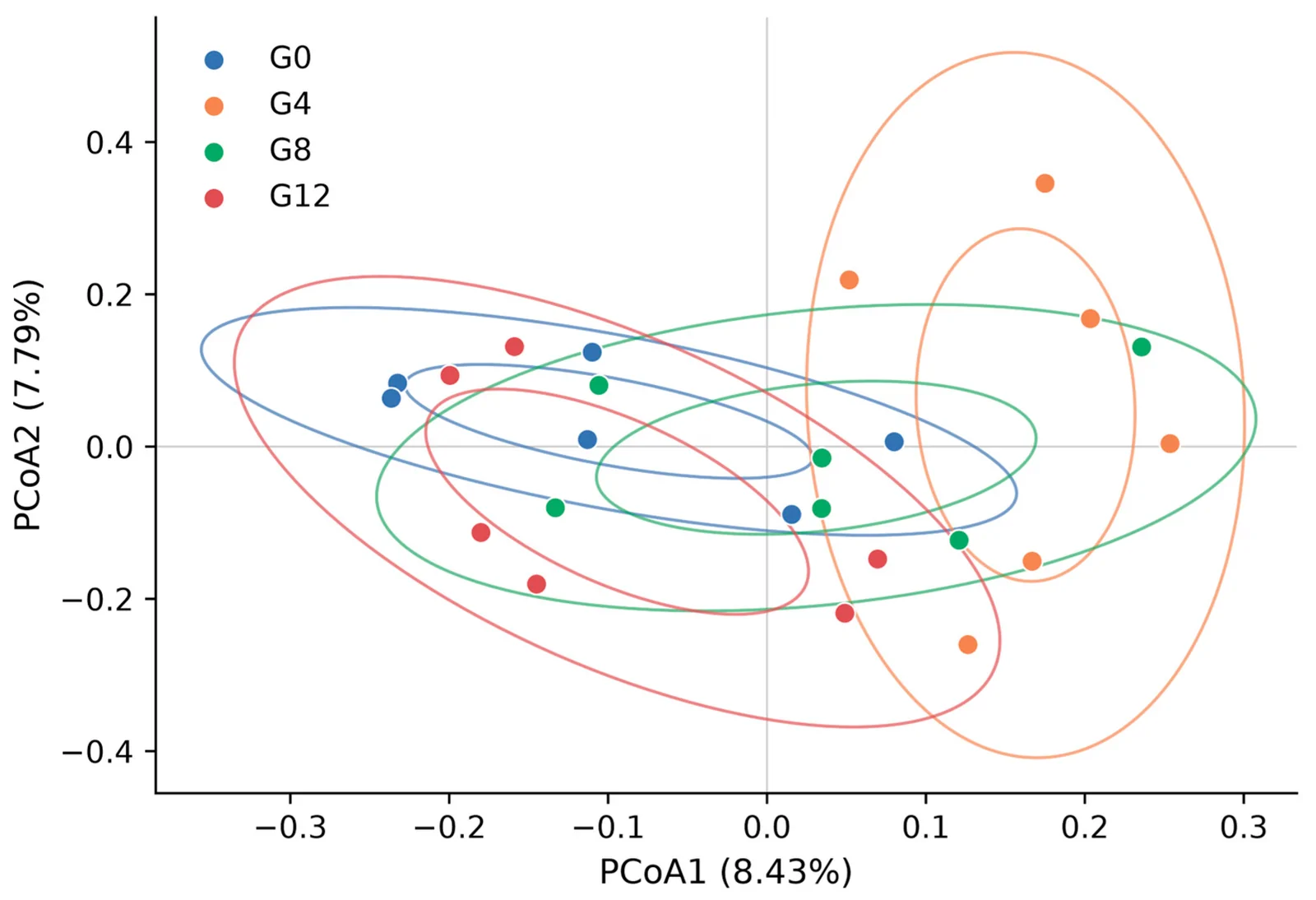
Principal coordinate analysis (PCoA) based on Bray–Curtis distances of the rumen bacterial community.

The PCoA based on Bray–Curtis distances (Figure 3) showed that PCoA1 and PCoA2 explained 8.43% and 7.79% of the community variation, respectively. Adonis revealed a significant overall difference in community structure among groups (*R*^2^ = 0.157, *p* = 0.002), although sample points overlapped considerably and inter-group separation was limited.

### 3.6. Differential Genera Among Groups

One-way ANOVA identified nine genera with nominal between-group variation (uncorrected *p* < 0.05; Table 5). UCG-004 was most abundant in G4 (1.11%) and significantly higher than in G0 (0.26%); norank_f__p-2534-18B5_gut_group was also highest in G4 (0.66%); *Syntrophococcus* was highest in G4 (0.21%) and lowest in G12 (0.06%), showing an overall decline with increasing WSSLs. However, after Benjamini–Hochberg FDR correction across all 210 genera tested, none of these genera remained significant (all q = 1.00); the patterns described below are interpreted as indicative trends rather than confirmed differential abundance.

### 3.7. Associations of Differential Genera with Fermentation and Growth and Slaughter Traits

**Figure 4:**
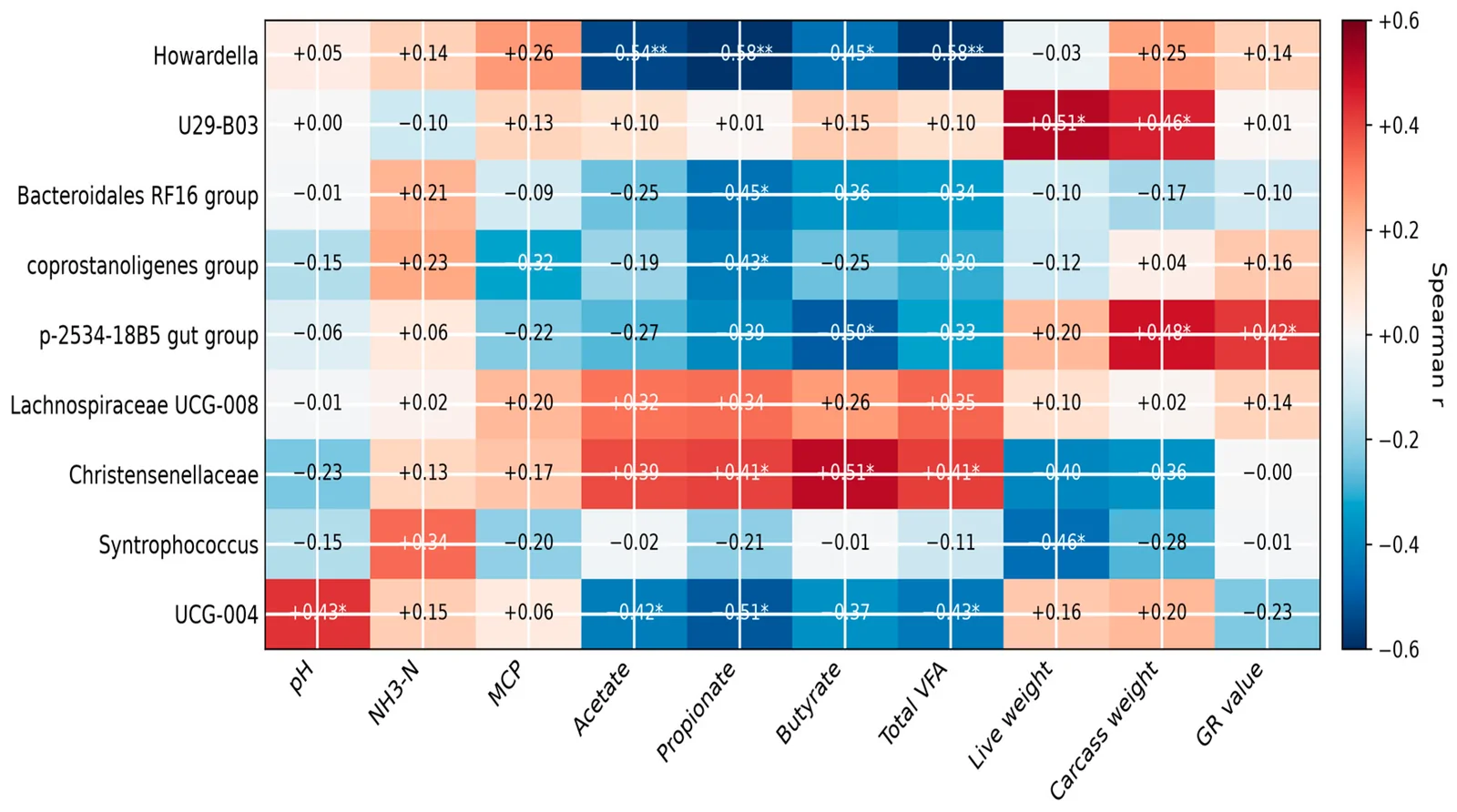
Spearman correlation heatmap between the nine nominal differential genera and ten traits (n = 24). Traits comprise seven fermentation variables (pH, NH3-N, MCP, acetate, propionate, butyrate and total VFA) and three growth/slaughter variables (live weight before slaughter, carcass weight and GR value) measured as described in Section 2.4 and Table 3. * *p* < 0.05, ** *p* < 0.01 (uncorrected). After Benjamini–Hochberg FDR correction across the 90 genus–trait tests, none remained significant (all q ≥ 0.134). Genus labels follow the abbreviated names defined in Table 5 (e.g., p-2534-18B5 gut group = norank_f__p-2534-18B5_gut_group).

Spearman correlation between the nominal differential genera and fermentation parameters (Table 6) showed nominal associations: *Howardella* was negatively correlated with acetate (r = −0.54, *p* < 0.01), propionate (r = −0.58, *p* < 0.01), butyrate (r = −0.45, *p* < 0.05) and total VFA (r = −0.58, *p* < 0.01). UCG-004 was correlated positively with pH (r = 0.43, *p* < 0.05) and negatively with acetate (r = −0.42, *p* < 0.05), propionate (r = −0.51, *p* < 0.05) and total VFA (r = −0.43, *p* < 0.05), while norank_f__Christensenellaceae was positively correlated with propionate (r = 0.41, *p* < 0.05), butyrate (r = 0.51, *p* < 0.05) and total VFA (r = 0.41, *p* < 0.05). The associations between the differential genera and growth and slaughter traits are shown in Figure 4: U29-B03 was positively correlated with live weight before slaughter (r = 0.51, *p* < 0.05) and carcass weight (r = 0.46, *p* < 0.05), norank_f__p-2534-18B5_gut_group was positively correlated with GR value (r = 0.42, *p* < 0.05) and carcass weight (r = 0.48, *p* < 0.05), and *Syntrophococcus* was negatively correlated with live weight before slaughter (r = −0.46, *p* < 0.05); correlations of the other genera with these traits were not significant (*p* > 0.05). In total, 19 of the 90 genus–trait tests reached *p* < 0.05 and three reached *p* < 0.01 before correction. However, after Benjamini–Hochberg FDR correction across the 90 genus–trait tests (9 genera × 10 traits; Table S2), none of these associations remained significant (all q ≥ 0.134); the U29-B03–live weight association, the strongest of the growth-related links, should be interpreted cautiously and confirmed in larger cohorts.

### 3.8. Predicted Functional Profiles

**Figure 5:**
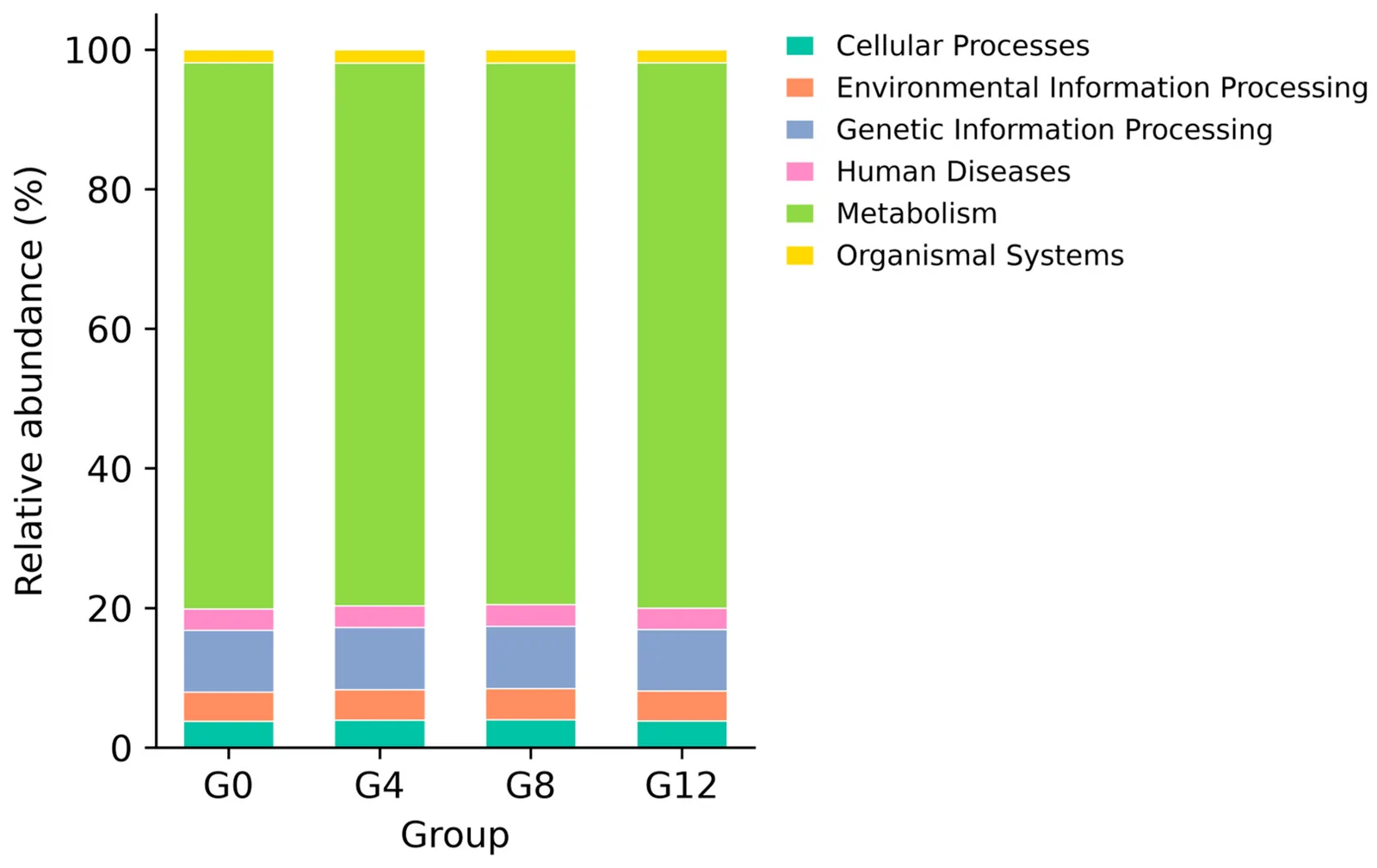
Relative abundance of KEGG level-1 functional categories in each group.

The weighted NSTI averaged 0.37 (range 0.30–0.42), indicating that the functional predictions were acceptable for reference. Across all groups, predicted genes were assigned mainly to Metabolism (about 78%), followed by Genetic Information Processing (about 8.8%), Environmental Information Processing (about 4.3%) and Cellular Processes (about 3.8%); none of the six level-1 categories differed significantly among groups (*p* = 0.15–0.93; Figure 5). At level 2, only two minor categories, antimicrobial drug resistance (*p* = 0.005) and cancer-specific types (*p* = 0.034), reached significance, but their relative abundances were below 1% and the differences were small without a dose–response; among COG categories, only replication, recombination and repair (category L) differed slightly (*p* = 0.01), by about 0.1 percentage point. Overall, the predicted functional profiles remained largely stable despite the changes in community composition.

## 4. Discussion

### 4.1. Effects of WSSLs on Rumen Fermentation Parameters

Ruminal pH is the most direct indicator of rumen homeostasis and the fermentation pattern; healthy sheep usually maintain pH between 6.2 and 7.0, and values persistently below 5.8 predispose them to subacute ruminal acidosis [8]. The pH values of 6.58–6.77 and the lack of significant differences in this study indicate that 4–12% WSSL inclusion did not disrupt the rumen acid–base balance, consistent with Duan et al. [14], who observed normal rumen fermentation and no obvious pH abnormality in Hu sheep fed wolfberry branches and leaves. VFAs are the end products of microbial carbohydrate fermentation, and their total concentration and molar composition reflect the extent of fermentation and the direction of energy utilization. The absence of significant differences in total VFA and in acetate, propionate and butyrate concentrations indicates that 4–12% WSSL inclusion did not markedly alter overall fermentation intensity.

The effects of wolfberry and other agricultural by-products on rumen fermentation are not uniform: residual black wolfberry fruit [17] and fermented rice husk powder [34] increased total VFA, whereas fermented corncob decreased total VFA in beef cattle while keeping acetate and propionate molar proportions and the acetate/propionate ratio stable [35]. These discrepancies may relate to the degree of lignification, whether the material was fermented and the inclusion level. In this study, WSSLs—the tender tissue left after sprout harvesting—was added directly in air-dried form. Total VFA concentration differed statistically among groups (*p* = 0.025), being higher in the control than in the WSSL groups, but the absence of a monotonic dose–response across 4%, 8% and 12% inclusion, together with the markedly larger variance in the control (SD 24.11 vs. 4.38–9.30 mmol/L), indicates that this difference reflects individual variation rather than a graded effect of WSSLs; Welch’s heteroscedastic ANOVA gave the same conclusion (*p* = 0.033). Community composition therefore changed without any consistent shift in overall fermentation intensity. The highest acetate/propionate ratio in G4 (2.74) suggests a tendency toward acetate-type fermentation at the low dose, possibly because the soluble fiber in WSSLs stimulated fibrolytic bacteria; Zhang et al. [18] likewise found that wolfberry by-products increased the activity of fibrolytic enzymes in grazing sheep. NH3-N reflects the dynamic balance between nitrogen degradation and microbial nitrogen capture, while MCP indicates the efficiency of ammonia use, both regulated by dietary nitrogen structure and microbial activity [36,37]. The lack of significant differences in NH3-N and MCP indicates that replacing corn straw with WSSLs did not markedly disturb the balance between rumen nitrogen degradation and immobilization. A constraint of the present design is that rumen fluid could be collected only once, immediately after slaughter and therefore after a 24 h fast, because sampling had to be combined with the routine commercial processing of the animals. Concentrations of volatile fatty acids, and to a lesser extent their molar proportions, are lower in the fasting state than in the fed state, so the data reported here characterize a standardized post-absorptive sampling point rather than the daily mean or the postprandial fermentation profile. The comparison among groups nevertheless remains valid, because all animals were sampled under identical conditions and after the same period on their respective diets.

### 4.2. Effects of WSSLs on Rumen Bacterial Diversity

Alpha diversity reflects community richness and evenness. The lack of significant differences in ACE, Chao1, Shannon and Simpson indices shows that WSSLs did not significantly alter the species diversity of the rumen microbiota. Henderson et al. [2] noted that the core rumen microbiome of adult ruminants is highly stable and functionally redundant, so alpha diversity usually remains stable when dietary change is limited; Ge et al. [38] and Li et al. [39] likewise found alpha diversity to be insensitive when dietary energy or diet type was changed in fattening sheep. Beta diversity reflects overall compositional differences. In our PCoA, Adonis was significant (*R*^2^ = 0.157, *p* = 0.002), but sample points overlapped heavily and the first two axes together explained only 16.2% of the variation, indicating that although the difference was statistically significant, actual separation was limited and the effect of WSSLs on overall community structure was mild. This resembles the findings of Ren et al. [40] comparing roughages in Tibetan sheep and of Chen et al. [41] supplementing lambs with probiotics and Chinese medicine polysaccharides, where plant-derived or probiotic additives caused only small, directional changes that inter-individual variation could partly mask. In contrast, Zhang et al. [25] observed clear community separation among different feeding patterns in Tan sheep, suggesting that distinct clustering in beta diversity appears only when dietary differences are large enough, which also explains the substantial overlap of the low- and medium-dose groups with the control.

### 4.3. Effects of WSSLs on Rumen Bacterial Community Composition

At the phylum level, Bacteroidota and Bacillota were the dominant phyla, together accounting for more than 85%, consistent with the rumen microbiota of Tan sheep [25], Tibetan sheep [40] and other ruminants. Bacteroidota are rich in carbohydrate-active enzymes and mainly degrade non-fibrous polysaccharides and proteins; their relative abundance tended to decrease with WSSL addition. Bacillota, which include many fibrolytic and butyrate-producing bacteria, showed the opposite pattern, and this reciprocal change indicates a shift in the substrate being fermented [3,36]. Fernando et al. [3] showed that the ratio of Bacteroidota to Bacillota changes markedly during the transition from forage to high-grain diets, and Wu et al. [36] found that dietary crude protein altered the phylum-level composition in Tibetan sheep.

At the genus level, *Xylanibacter* was the most abundant genus (15.85–18.18%). This genus belongs to Prevotellaceae and has a strong capacity to degrade xylan and hemicellulose; Ren et al. [40] also detected high abundances of *Xylanibacter* and *Succiniclasticum* in Tibetan sheep fed wheat straw. Its decline with increasing WSSLs may reflect the lower hemicellulose content of WSSLs relative to corn straw and the corresponding reduction in available substrate. *Treponema* increased in the supplemented groups; its members participate in fiber and pectin degradation and can act synergistically with other fibrolytic bacteria, so their expansion reflects adaptation to the changed fiber substrate. Genera involved in succinate metabolism and propionate production, such as *Saccharofermentans* and *Succiniclasticum*, remained relatively stable, in line with the unchanged total VFA and acetate/propionate ratio. The early colonization of core functional rumen bacteria is strongly regulated by diet [23], whereas the microbiota of adult fattening sheep is already stabilized, which may largely explain the modest overall community change in this study.

### 4.4. Interpretation of the Genera–Trait Associations in the Context of WSSL Supplementation

Linking differential genera to fermentation and growth phenotypes helps interpret the action of an additive from a microbial perspective [42]. Of the nine nominal differential genera, *Howardella* was negatively correlated with acetate, propionate, butyrate and total VFA. This genus belongs to Erysipelotrichaceae, and some members participate in amino acid deamination and sugar metabolism; its increased abundance accompanied by lower VFAs suggests that it may compete for or consume fermentation intermediates, although its exact function requires metagenomic evidence. UCG-004 was correlated positively with pH and negatively with acetate, propionate and total VFA. This group belongs to Ruminococcaceae and tends to be enriched when pH is high and fiber fermentation is suppressed, consistent with the general pattern of rumen fermentation [8]. In the present study, however, none of the nine nominal differential genera survived FDR correction, and none of their correlations with fermentation or growth traits did either, so the observations below are exploratory.

The norank Christensenellaceae were nominally positively correlated with propionate, butyrate and total VFA. Christensenellaceae are considered closely related to host energy harvest and feed efficiency; Wang et al. [42] found an association between rumen microbiota and intramuscular fat deposition (marbling grade) in Hu sheep, and the positive correlation of this family with acid production in our study may indicate a beneficial role in rumen energy utilization, although this association did not survive FDR correction and is reported as nominal only.

For growth and slaughter traits, U29-B03 was nominally positively correlated with live weight before slaughter and carcass weight, norank_f__p-2534-18B5_gut_group with GR value and carcass weight, while *Syntrophococcus* was negatively correlated with live weight before slaughter. *Syntrophococcus* participates in cross-feeding for interspecies hydrogen transfer and acetate formation, and changes in its abundance may affect rumen hydrogen use and fermentation direction. U29-B03 and p-2534-18B5_gut_group have limited taxonomic information, and their positive associations with meat-producing traits may not depend on the VFA pathway but rather on microbial protein synthesis or other metabolic routes; Kang et al. [43] likewise found that some unclassified genera were related to carcass traits when studying the effects of arginine on rumen bacterial composition in sheep. Importantly, because these growth associations were nominal and did not survive FDR correction, they cannot be taken as evidence that WSSLs promote growth through these taxa.

Ju et al. [10,11] showed that an appropriate dose of *L. barbarum* polysaccharides increased daily gain and protein utilization in lambs, and Ma et al. [21] found that wolfberry residue affected feeding behavior and digestibility in Tan sheep. These studies support the potential of wolfberry by-products to benefit ruminant production; however, in the present study the associations between the nominal differential genera and growth traits were nominal and did not survive FDR correction, so they do not provide evidence that WSSLs affect growth through specific taxa. However, because this 16S rRNA gene-based association analysis cannot establish causality, the metabolic functions of the relevant genera and the underlying mechanisms require further verification using metagenomics, metabolomics and isolation and culture.

### 4.5. Stability of Predicted Functions and Functional Redundancy

A notable finding is that WSSLs changed the relative abundance of several taxa only nominally (none survived FDR correction across all 210 genera tested) and yielded a significant Adonis result, yet the predicted functional profiles were almost unchanged, with Metabolism consistently accounting for about 78% and no level-1 category differing among groups. This decoupling of taxonomic composition from functional output is characteristic of functional redundancy in the rumen microbiome: different taxa carry overlapping gene sets, so moderate compositional turnover can be buffered without loss of core metabolic capacity [2]. Similar composition-shifted but function-stable patterns have been reported in sheep and cattle given modest dietary changes [35,38]. The few level-2 and COG categories that reached statistical significance involved very low-abundance functions and lacked a dose–response, and are therefore more likely statistical fluctuations than meaningful biological shifts. Together with the stable pH and VFA profiles, these results indicate that 4–12% WSSL inclusion reshaped part of the community structure while preserving overall rumen metabolic function, further supporting the safety of WSSLs as an unconventional roughage. Because PICRUSt2 predictions are inferred rather than measured and the NSTI values were moderate (0.37), pathway-level conclusions should be confirmed by metagenomic or metatranscriptomic sequencing. Finally, two further features of the design should be borne in mind. Because the four diets were balanced to be isonitrogenous and isoenergetic, small adjustments to corn, wheat bran, corn germ meal, rapeseed meal, cottonseed meal and DDGS necessarily accompanied increasing levels of WSSLs; strictly speaking, therefore, the comparison is between four nutritionally balanced diets rather than between diets differing in WSSLs alone, and this should be considered when attributing an effect to WSSLs per se. In addition, the animals within each group were housed in three pens of four; because all animals within a pen received the same diet and every measurement was made on individual animals, the individual animal was treated as the experimental unit and any pen effect is subsumed in the residual term.

## 5. Conclusions

Under the conditions of this study, dietary supplementation with 4–12% WSSLs did not affect ruminal pH, NH3-N, MCP or bacterial alpha diversity, whereas total VFA concentration differed among groups in fattening Tan sheep. The rumen bacterial community showed only limited, non-significant shifts in individual taxon abundance: among 210 genera tested, nine displayed nominal between-group variation (lowest uncorrected *p* = 0.011), but none remained significant after Benjamini–Hochberg FDR correction (all q = 1.00). Exploratory correlations between these nominal genera and fermentation or growth and slaughter traits were likewise not significant after FDR correction (all q ≥ 0.134) and are reported as nominal only. Beta-diversity analysis revealed a statistically significant yet biologically mild reorganization (Adonis R^2^ = 0.157, *p* = 0.002) with substantial inter-group overlap, and the predicted functional profiles were largely unchanged, reflecting functional redundancy of the rumen microbiome. Overall, WSSLs can be used as an unconventional feed resource for fattening Tan sheep without adversely affecting ruminal pH, nitrogen metabolism or growth performance; its inclusion reshaped the community only marginally and preserved core rumen function, supporting its safety at appropriate inclusion levels.

## Figures and Tables

**Table 1:** Composition and nutrient levels of the experimental diets (dry matter basis), %.

Items	G0	G4	G8	G12
Corn	34	35	35	35
Wheat bran	6	6	5	6
Corn germ meal	5	5	6	5
Rapeseed meal	5	4	4	4
Cottonseed meal	4	5	4	3
DDGS	6	5	6	7
Corn straw	20	16	12	8
Wolfberry sprout stems and leaves	0	4	8	12
Corn cob	15	15	15	15
Extruded urea	1	1	1	1
Calcium hydrogen phosphate	1	1	1	1
Sodium chloride	1	1	1	1
Limestone	1	1	1	1
Premix 1	1	1	1	1
Total	100	100	100	100
Nutrient levels				
Metabolizable energy, MJ/kg	9.90	9.97	10.60	10.00
Crude protein, %	14.80	14.90	14.80	15.00

1 The premix provided the following per kg of diet: vitamin A 15,000 IU, vitamin D_3_ 5000 IU, vitamin E 50 IU, Fe 60 mg, Cu 10 mg, Mn 40 mg, Zn 60 mg, Se 0.3 mg, I 0.5 mg, Co 0.2 mg. DDGS, distillers dried grains with solubles; ME, metabolizable energy; CP, crude protein.

**Table 2:** Rumen fermentation parameters of sheep in different groups.

Item	G0	G4	G8	G12	*p*-Value
pH	6.58 ± 0.26	6.77 ± 0.06	6.77 ± 0.22	6.75 ± 0.16	0.266
NH3-N, mg/100 mL	2.22 ± 0.49	2.31 ± 0.71	2.12 ± 0.64	1.89 ± 0.36	0.608
MCP, mg/100 mL	0.77 ± 0.20	0.69 ± 0.08	0.74 ± 0.13	0.79 ± 0.16	0.674
Acetate, mmol/L	29.47 ± 10.45	17.72 ± 4.86	22.22 ± 4.63	23.81 ± 2.02	0.032
Propionate, mmol/L	15.60 ± 10.52	6.56 ± 1.85	9.09 ± 2.73	10.71 ± 1.78	0.067
Butyrate, mmol/L	6.92 ± 3.48	2.98 ± 0.55	3.82 ± 1.45	3.68 ± 0.53	0.01
Valerate, mmol/L	0.94 ± 0.34	0.54 ± 0.10	0.69 ± 0.26	0.64 ± 0.13	0.039
Total VFA, mmol/L	56.27 ± 24.11	30.15 ± 7.02	38.92 ± 9.30	41.53 ± 4.38	0.025
Acetate/propionate	2.11 ± 0.48	2.74 ± 0.57	2.54 ± 0.59	2.26 ± 0.28	0.153

Values are means ± standard deviation (SD); n = 6 per group. NH3-N, ammonia nitrogen; MCP, microbial crude protein; A/P, acetate-to-propionate ratio.

**Table 3:** Growth performance and slaughter traits of Tan sheep fed diets containing different levels of WSSLs (n = 6 per group).

Item	G0	G4	G8	G12	*p*-Value
Initial body weight, kg	22.57 ± 1.76	23.25 ± 1.83	21.70 ± 1.38	23.07 ± 1.58	0.387
Final body weight, kg	38.53 ± 1.68	38.77 ± 1.89	40.58 ± 1.60	40.09 ± 1.70	0.144
Average daily gain, g/d	270.6 ± 15.2	263.1 ± 35.9	320.1 ± 32.5	288.6 ± 23.6	0.010
Live weight before slaughter, kg	35.43 ± 2.55	37.27 ± 1.77	38.54 ± 1.50	39.09 ± 1.11	0.011
Carcass weight, kg	18.00 ± 0.98	19.17 ± 1.20	19.78 ± 0.72	20.05 ± 1.17	0.013
GR value, mm	16.12 ± 2.55	15.86 ± 2.92	15.80 ± 1.66	17.71 ± 2.42	0.493

Values are means ± standard deviation (SD); n = 6 per group. ADG, average daily gain; GR, grading ruler value measured between the 12th and 13th ribs; live weight before slaughter was recorded after a 24 h fast; carcass weight excludes the skin, head, feet and internal organs but retains the kidneys and perirenal fat; the GR value is the tissue thickness between the 12th and 13th ribs at 11 cm from the dorsal midline.

**Table 4:** Alpha diversity indices of the rumen bacterial community.

Item	G0	G4	G8	G12	*p*-Value
ACE	660.49 ± 72.88	617.49 ± 47.82	657.16 ± 74.33	694.07 ± 62.99	0.353
Chao1	660.36 ± 72.83	617.33 ± 47.84	656.94 ± 74.34	693.85 ± 62.95	0.354
Shannon	5.39 ± 0.22	5.35 ± 0.16	5.40 ± 0.23	5.38 ± 0.21	0.978
Simpson	0.01 ± 0.01	0.01 ± 0.00	0.01 ± 0.00	0.01 ± 0.00	0.782
Coverage	1.00 ± 0.00	1.00 ± 0.00	1.00 ± 0.00	1.00 ± 0.00	0.121

Values are means ± standard deviation (SD); n = 6 per group. ACE, abundance-based coverage estimator; Chao1, Chao1 richness estimator; Shannon and Simpson, diversity indices; Coverage, Good’s coverage of the sequencing depth.

**Table 5:** Relative abundance of nine nominal differential genera among groups, %. Uncorrected *p* < 0.05; after Benjamini–Hochberg FDR correction across all 210 genera, none remained significant (all q = 1.00).

Differential Genus	G0	G4	G8	G12	*p*-Value
*Howardella*	0.007 ± 0.009	0.031 ± 0.014	0.021 ± 0.014	0.010 ± 0.009	0.011
U29-B03	0.268 ± 0.040	0.240 ± 0.071	0.531 ± 0.174	0.395 ± 0.243	0.015
Bacteroidales RF16 group	0.222 ± 0.164	0.626 ± 0.419	0.191 ± 0.195	0.188 ± 0.149	0.019
coprostanoligenes group	0.605 ± 0.215	1.103 ± 0.401	0.634 ± 0.365	0.653 ± 0.106	0.025
p-2534-18B5 gut group	0.034 ± 0.037	0.663 ± 0.628	0.233 ± 0.197	0.228 ± 0.159	0.029
Lachnospiraceae UCG-008	0.153 ± 0.079	0.062 ± 0.037	0.081 ± 0.068	0.140 ± 0.023	0.030
Christensenellaceae (norank)	0.052 ± 0.024	0.025 ± 0.018	0.019 ± 0.015	0.032 ± 0.016	0.035
*Syntrophococcus*	0.123 ± 0.089	0.214 ± 0.112	0.097 ± 0.078	0.064 ± 0.055	0.038
UCG-004	0.260 ± 0.105	1.114 ± 0.534	0.798 ± 0.745	0.746 ± 0.316	0.047

Values are means ± standard deviation (SD); n = 6 per group. G0, G4, G8 and G12, diets in which 0%, 4%, 8% and 12% of corn straw was replaced by WSSLs, respectively; norank_f__, a family-level lineage that could not be assigned to a named genus. Full SILVA names of the abbreviated taxa: norank_f__Bacteroidales_RF16_group (Bacteroidales RF16 group), [Eubacterium]_coprostanoligenes_group (coprostanoligenes group), norank_f__p-2534-18B5_gut_group (p-2534-18B5 gut group) and norank_f__Christensenellaceae (Christensenellaceae, norank); Lachnospiraceae_UCG-008 is abbreviated as Lachnospiraceae UCG-008.

**Table 6:** Spearman correlation coefficients between nominal differential genera and fermentation parameters. * *p* < 0.05, ** *p* < 0.01 (uncorrected). After Benjamini–Hochberg FDR correction across the 90 genus–trait tests, none remained significant (all q ≥ 0.134; full 90-test matrix in Table S2).

Differential Genus	pH	MCP	NH3-N	Acetate	Propionate	Butyrate	Total VFA
*Howardella*	+0.05	+0.26	+0.14	−0.54 **	−0.58 **	−0.45 *	−0.58 **
U29-B03	+0.00	+0.13	−0.10	+0.10	+0.01	+0.15	+0.10
Bacteroidales RF16 group	−0.01	−0.09	+0.21	−0.25	−0.45 *	−0.36	−0.34
coprostanoligenes group	−0.15	−0.32	+0.23	−0.19	−0.43 *	−0.25	−0.30
p-2534-18B5 gut group	−0.06	−0.22	+0.06	−0.27	−0.39	−0.50 *	−0.33
Lachnospiraceae UCG-008	−0.01	+0.20	+0.02	+0.32	+0.34	+0.26	+0.35
Christensenellaceae (norank)	−0.23	+0.17	+0.13	+0.39	+0.41 *	+0.51 *	+0.41 *
*Syntrophococcus*	−0.15	−0.20	+0.34	−0.02	−0.21	−0.01	−0.11
UCG-004	+0.43 *	+0.06	+0.15	−0.42 *	−0.51 *	−0.37	−0.43 *

Spearman correlation coefficients; n = 24. * *p* < 0.05, ** *p* < 0.01 (uncorrected). NH3-N, ammonia nitrogen; MCP, microbial crude protein; total VFA, total volatile fatty acids. Genus names follow Table 5: norank_f__Bacteroidales_RF16_group (Bacteroidales RF16 group), [Eubacterium]_coprostanoligenes_group (coprostanoligenes group), norank_f__p-2534-18B5_gut_group (p-2534-18B5 gut group) and norank_f__Christensenellaceae (Christensenellaceae, norank).

## Data Availability

The raw 16S rRNA gene sequencing data have been deposited in the NCBI Sequence Read Archive (SRA) under BioProject accession PRJNA1549111 (BioSample accessions SAMN64377473–SAMN64377496; SRA run accessions SRR41323158–SRR41323181) and will be made publicly available upon publication. Sample-level metadata are additionally provided as Table S3 (SRA_BioSample_metadata.tsv) in the Supplementary Materials.

## Supplementary Materials

The following supporting information can be downloaded at: https://www.mdpi.com/article/10.3390/ani16213430/s1, Table S1: ARRIVE 2.0 Essential 10 checklist detailing the reporting of this animal study. Table S2: Full 90-test genus–trait Spearman correlation matrix (9 genera × 10 traits, n = 24), with uncorrected p-values and Benjamini–Hochberg FDR-adjusted q-values. Table S3: Sample-level SRA/BioSample metadata (SRA_BioSample_metadata.tsv).
