# Supplementary material for: Effects of Dietary Wolfberry Sprout Stems and Leaves on the Rumen Bacterial Community of Fattening Tan Sheep and Its Association with Fermentation and Growth Traits
Source: Animals (Basel). 2026 Oct 30;16(21):3430. doi: 10.3390/ani16213430 (PMC13761422; doi:10.3390/ani16213430)
Supplement: Supplementary file 1 [file animals-16-03430-s001.zip › Table S1_ARRIVE_2.0_Essential_10_checklist.pdf]

# Table S1. ARRIVE 2.0 Essential 10 checklist

*Manuscript: animals-4621870 (Animals, MDPI) — Effects of Dietary Wolfberry Sprout Stems and Leaves on the Rumen Bacterial Community of Fattening Tan Sheep and Its Association with Fermentation and Growth Traits*

| Item | Recommendation (ARRIVE 2.0) | Response in manuscript (section) |
|---|---|---|
| **1. Study design** | a. The groups being compared, including control groups. If no control group has been used, the rationale should be stated. | Four groups: G0 (basal diet, control), G4, G8 and G12, in which 4%, 8% and 12% of the corn straw in the basal diet was replaced by WSSL (Section 2.2; Table 1). |
| | b. The experimental unit (e.g. a single animal, litter, or cage of animals). | The individual animal was the experimental unit for all measurements (Section 2.2). |
| **2. Sample size** | a. Specify the exact number of experimental units allocated to each group, and the total number in each experiment. Also indicate the total number of animals used. | 48 Tan rams were allocated to four groups of 12; 6 sheep per group (24 in total) were selected for slaughter, rumen sampling and sequencing (Section 2.2). |
| | b. Explain how the sample size was decided. Provide details of any a priori sample size calculation, if done. | No a priori sample-size calculation is reported; 12 animals per group were used, and the 6 animals closest to the group mean body weight were selected for slaughter and sequencing (Section 2.2). |
| **3. Inclusion and exclusion criteria** | a. Describe any criteria used for including and excluding animals (or experimental units) during the experiment, and data points during the analysis. Specify if these criteria were established a priori. If no criteria were set, state this explicitly. | Inclusion: healthy 3-month-old weaned Tan rams with similar body weight (22.75 ± 0.52 kg) and body condition (Section 2.2). At the end of the trial, six sheep per group with body weight closest to the group mean were selected for slaughter and sequencing. No criteria were pre-specified for excluding data points. |
| | b. For each experimental group, report any animals, experimental units or data points not included in the analysis and explain why. If there were no exclusions, state so. | No animals or data points were excluded from the analyses (Section 2.7; Tables 2–5). |
| | c. For each analysis, report the exact value of n in each experimental group. | n = 6 per group for all fermentation, growth, slaughter and 16S analyses; n = 24 for Spearman correlations (Section 2.7; Tables 2–6). |
| **4. Randomisation** | a. State whether randomisation was used to allocate experimental units to control and treatment groups. If done, provide the method used to generate the randomisation sequence. | Yes. Sheep were randomly assigned to four groups of 12 (Section 2.2). The method used to generate the allocation sequence is not specified in the manuscript. |
| | b. Describe the strategy used to minimise potential confounders such as the order of treatments and measurements, or animal/cage location. If confounders were not controlled, state this explicitly. | All sheep were housed indoors and tethered under the same management and feeding regime (Section 2.2); no additional strategy to control confounders is described. |
| **5. Blinding** | Describe who was aware of the group allocation at the different stages of the experiment (during the allocation, the conduct of the experiment, the outcome assessment, and the data analysis). | Blinding was not applied: the four experimental diets were visibly different among groups, so the trial was conducted in an open-label manner; group allocation was known to the personnel involved in animal management, feeding and sampling (Section 2.2). |
| **6. Outcome measures** | a. Clearly define all outcome measures assessed (e.g. cell death, molecular markers, or behavioural changes). | Rumen fermentation (pH, $NH_3$-N, MCP, VFA profile, acetate/propionate ratio); growth (initial and final body weight, ADG); slaughter (live weight before slaughter, carcass weight, GR value); rumen bacterial community (α-diversity: ACE, Chao1, Shannon, Simpson, coverage; β-diversity: Bray–Curtis PCoA; relative abundances and differential genera); predicted functions (PICRUSt2; KEGG) (Sections 2.4–2.6, 3). |
| | b. For hypothesis-testing studies, specify the primary outcome measure, i.e. the outcome measure that was used to determine the sample size. | The rumen bacterial community and its association with fermentation and growth traits is the primary focus; no separate sample-size-determining primary outcome was pre-specified (Sections 1, 2.2). |
| **7. Statistical methods** | a. Provide details of the statistical methods used for each analysis, including software used. | One-way ANOVA ($Y_{ij} = \mu + T_i + e_{ij}$) with Duncan's multiple range test; Benjamini–Hochberg FDR adjustment; Bray–Curtis PCoA with Adonis (PERMANOVA, 999 permutations); Spearman rank correlation (n = 24); R 4.2.1 and Python 3.10 (Section 2.7). |
| | b. Describe any methods used to assess whether the data met the assumptions of the statistical approach, and what was done if the assumptions were not met. | The manuscript does not explicitly report assessment of ANOVA assumptions (normality, homogeneity of variance) or remedial measures (Section 2.7). |

| | | |
|---|---|---|
| **8. Experimental animals** | a. Provide species-appropriate details of the animals used, including species, strain and substrain, sex, age or developmental stage, and, if relevant, weight. | Species: sheep (Ovis aries); breed: Tan sheep; sex: male; age: 3 months (weaned); body weight: 22.75 ± 0.52 kg (Section 2.2). |
| | b. Provide further relevant information on the provenance of animals, health/immune status, genetic modification status, genotype, and any previous procedures. | Provenance: Nuanquan Sheep Farm of Ningxia Nongken Beef and Mutton Food Co., Ltd.; healthy animals; no genetic modification; no previous experimental procedures (Section 2.2). |
| **9. Experimental procedures** | a. What was done, how it was done and what was used. | Diets (Table 1); WSSL preparation (Section 2.3); rumen sampling and fermentation assays (Section 2.4); DNA extraction and 16S rRNA gene sequencing (Section 2.5); PICRUSt2 functional prediction (Section 2.6). |
| | b. When and how often. | 10-day adaptation followed by a 60-day experimental period; fed twice daily at 08:00 and 17:00; 24-h fast and 2-h water deprivation before slaughter (Sections 2.2, 2.4). |
| | c. Where (including detail of any acclimatisation periods). | Nuanquan Sheep Farm of Ningxia Nongken Beef and Mutton Food Co., Ltd., Ningxia, China; 10-day adaptation period (Sections 2.1, 2.2). |
| | d. Why (provide rationale for procedures). | To evaluate the effects of replacing corn straw with WSSL on the rumen bacterial community and its association with fermentation and growth traits (Section 1). |
| **10. Results** | a. Summary/descriptive statistics for each experimental group, with a measure of variability where applicable (e.g. mean and SD, or median and range). | Mean ± SD for each group are reported in Tables 2–5; n = 6 per group (Section 3). |
| | b. If applicable, the effect size with a confidence interval. | Effect sizes with confidence intervals are not reported; group differences are presented as mean ± SD with p-values (Tables 2–6). |

Section numbers refer to the revised manuscript. ADG, average daily gain; GR, grading ruler value; MCP, microbial crude protein; NH3-N, ammonia nitrogen; VFA, volatile fatty acid; WSSL, wolfberry sprout stems and leaves; SD, standard deviation.
